# Book Reviews

**Published:** 1818-04

**Authors:** 


### CRITICAL ANALYSIS

### OF RECENT PUBLICATIONS,

IN THE

### DIFFERENT BRANCHES OF PHYSIC, SURGERY, AND MEDICAL PHILOSOPHY.

---

*Cases in Surgery. Part II. Of Diseased Prepuce and Scrotum; illustrated with Etchings.* By WILLIAM WADD, Esq. Surgeon Extraordinary to his Royal Highness the Prince Regent. 4to. 11s. pp. 31. Callow, London.

WE are much pleased at the sight of another Number from this accurate physiologist, and hope that the custom of etching will be more generally adopted by surgeons who only can judge of the importance of attending to the minuter parts. The present plates very much surpass the former in softness and technical finish, though they cannot in correctness. They are twelve in number: we shall give a short account of each. The first contains three forms of diseased prepuce, boldly and correctly delineated. The introduction, part of which we shall transcribe, will show the intention of the author.

"Probably the use of the prepuce is to protect the delicate thin skin of the glans in animals who have no artificial clothing, and, of course, in man in his savage state. That it is not necessary for the purposes of the organ of which it makes a part, is evident by the variety in its natural figure, and by the frequency of circumcision. On this account, any impediment it may offer to the natural functions ought to be speedily removed.

"But, however unimportant this covering of the glans penis, or præputium, may seem, it is frequently, from malformation or disease, the cause of much inconvenience to the functions of that organ, and sometimes of very serious impediment to the ordinary functions of the bladder.

"In a work, therefore, intended to represent most of the discases which may be relieved by the surgeon, those of the prepuce naturally fall under this division.

"Turner, though he includes the diseases of the prepuce among those of the skin, found it necessary to devote a chapter to two complaints peculiar to this part; namely,—*Phymosis* and *Paraphymosis.*

"Phymosis is a contraction of the orifice of the prepuce, which prevents its being retracted or withdrawn, in the manner before described. It is sometimes congenital; and the inconvenience arising from it is now so well understood, that the difficulty of voiding the urine, occasioned by this state of the parts, is generally remedied by operation, before the patient attains the age of puberty. Where this has been neglected, diseases of the urethra and bladder have been the consequence.

"Paraphymosis is a condition of the prepuce, in which it is already retracted, but cannot be returned to its original form. In this case it produces the effect of a ligature round the basis of the glans, and is, on that account, by some called *Periphymosis.*

"When the phymosis is complete, no part of the glans, nor even the orifice of the urethra, can be discovered. This occurs sometimes in advanced age, apparently from a gradual shrinking of the penis; after which, the projecting orifice of the prepuce contracts to such a degree as to hinder the water from passing, even after it has escaped from the urethra. Hence, the whole cavity of the prepuce becomes filled with urine; a small quantity of which, constantly covering the glans, deposits a calculous crust, assuming the figure of that part.

"When the prepuce is thin, a division of the part with a phymosis knife, or curved bistoury, generally gives relief. When the prepuce is thicker, some have proposed an operation similar to that for the hare-lip, in order to obviate the deformity, from a separation of the two lamina of the skin: but, as the part is not exposed to view, this appears unnecessary.

"Others prefer circumcision; compressing as much of the prepuce as is necessary within the blades of the forceps, and cutting it off with one stroke of the knife. In hot climates, where the inhabitants are most exposed to the inconvenience of morbid secretions from this part, Christians, as well as Jews, submit to this operation, according to an observation of Guido de Cauliaco,—'Propterea quod non congregantur sordities in radis balani et calefacerent ipsum.'

"Fallopius proposes a gradual dilatation, without any incision; which, in some cases, may succeed.

"When phymosis has existed a long time, adhesions take place between the glans and the prepuce, which cannot always be separated.

"Sometimes, if the pus formed between the prepuce and glans cannot escape by the orifice, ulceration takes place through the prepuce, by which the glans protrude, and the lower part of the prepuce is thrown to one side, resembling the finger of a glove, open at its extremity.

"The congenital phymosis in children sometimes goes off in adult age, the prepuce enlarging in greater proportion than the glans. An operation should not, therefore, be advised, unless other circumstances require it; though, to judge by my own experience, this natural cure of the constriction is of rare occurrence."

Two plates follow, shewing the effects of phymosis on the urethra. Four specimens of disease in that membrane are given, where the general history affords no other probable cause than long continued resistance to its expulsitory power. The upper portion of urethra in plate II. is a fungus, which in former times would have been denominated a caruncle. The lower portion exhibits a membranous band across the urethra, behind which is an oval ulceration. In many parts of these urethræ were appearances of disease and inflammation, which, not producing alteration in the form and figure of the part, cannot be delineated by the pencil.

The two succeeding plates represent the state of the foreskin, where urine, each time it was voided, filling the cavity, and partly resting on the glans, gradually induced a deposition of calculous matter on that organ. The natural opening was obliterated by inflammation, and the urine could only escape with difficulty through ulcerations on the side and under part. Here circumcision was necessary, and, when performed, exposed a margin of fungus and calculous incrustation: the latter was easily removed, and the fungus afterwards by the application of caustic.

The sixth plate gives a representation of the effects of an abscess between the two layers of the prepuce, with suitable remarks.

The seventh is an etching of a cancerous prepuce. Of this the author speaks with some caution; and, as the subject has been so recently before us, we shall do him the credit to show, by the following extract, that he, at least, is not among the number of those who are unmindful of their predecessors.

"Here were many of the characteristics of carcinoma. A sordid, sanious, fœtid discharge. The erosions betwixt the fungi bled from time to time, and the serrated, indurated, retorted edge, presented the external marks of cancer.

"Many apparently trifling diseases of these parts, whether arising from mal-formation or want of cleanliness, derive their importance from their situation. Of this class are herpetic affections of the skin, and ulceration from the lodgements of the mucous secretion between the contracted prepuce and the penis. These, when remarked with sufficient accuracy, may be always distinguished from syphilis. It is not so easy to describe them by an appropriate character. Till Mr. Hunter's time, there was no difficulty, because they were all called venereal. A celebrated French author said formerly,—'On peut assurer que quand trente mille hommes combattent en bataille rangée contre des troupes égales en nombre, il y a environ Vingt mille Vérolés de chaque côté;'—but *grande verole, lues*, and *pox*, are now obsolete terms. Writers have been ashamed to confound what Mr. Hunter separated with so much accuracy; and, by slow degrees, the true venereal ulcer has been tolerably well ascertained. But a new language has been introduced, and threatens to confound all other distinctions. We had long been accustomed to the word *proteiform*, which proved a most convenient salvo, till Mr. Hunter shewed the uniformity of nature in this as in all other causes and effects. Pseudo-syphilis now removes us a single step, and no more, in our labyrinth; but, what is much worse, it proves an apology for resting, instead of proceeding and marking our way. Mr. Hunter, though he gave no names to the other numerous complaints of this organ, yet described them with accuracy, so that, when we meet with them, we recognise what we have seen in his writings. If we are still at a loss for names, there is reason to believe most of them may be found in Celsus: see his chapter, *De obscœnarum partium vitiis.* Such is not, however, the case with the two following.

The plate to which the close of the foregoing extract refers, gives a representation of the enlarged prepuce, so common in the West-India islands, particularly in Barbadoes, as to be almost endemic. This, as the author observes, is, by the moderns, termed elephantiasis, when seated in the foot and leg. The other remarks are very judicious, and the references to the best authors not less copious. To render the illustrations more perspicuous, this case is followed by a figure of the penis under the elephantiasis of Aretæus, in which that organ appears somewhat retracted within the pubes, which is free from hair, and the prepuce appears elongated by the retraction and, probably, wasting of the glans within it.

"Aretæus (observes Mr. Wadd) is allowed to be the first author who notices this disease, and his description has been copied by every subsequent writer, till our own days. For the most part, it is admitted to be correct. If it is deficient in the parts under consideration, it should be remembered, that the author acknowledges his fear of contagion, which probably prevented his closer examination: nor is it unlikely that, when he speaks of the salaciousness of these unhappy creatures, he only relates a vulgar error.

"A case of this kind lately occurred in St. Bartholomew's Hospital, and is related in the Medico-Chirurgical Transactions, vol. vi. I did not see it, but the following is the description given by Mr. Lawrence :—'Not only had the development of the generative organs been arrested from the time when the disease broke out, but they had actually undergone diminution and decay. The scrotum was shrivelled, and seemed empty ; the testes could with difficulty be felt ; they were soft, and about the size of small horse-beans."

The present number introduces the Affections of the Scrotum, and closes with two most interesting plates of a disease, we believe, not before accurately represented on paper, and at present particularly requiring the attention of the faculty, on account of parliamentary enquiry. This is the chimney-sweepers' cancer, of which four representations are given in its different forms. We cannot admit space to enlarge on the author's pathology of the disease, which is to us not less satisfactory than perspicuous.

Such are the contents of this number, of which, by several hints interspersed, we expect a continued series. We sincerely hope Mr Wadd will improve the leisure of the succeeding summers, as he has done the last. During the winter (to speak professionally) he is probably much better employed.

---

*Obervations relative to the Treatment, by Sir William Adams, of the Ophthalmic Cases of the Army.* By John Vetch, M.D. Physician to the Forces, Member of the Medical and Chirurgical Society of London. and of the Royal Medical Society of Edinburgh. 8vo. pp. 26. Callow, London.

This little pamphlet contains some passages which we shall only transcribe. The character of Dr. Vetch, as an able writer, is known to most of our readers ; and the very extensive practice with which he has been so long entrusted at the army depôt, is not a little to his credit.

The work commences with a few very appropriate remarks on the difference between an epidemic ophthalmia among girls at the well-regulated Military Asylum, and soldiers in the highest health, at the most vigorous period of life, often-times artificially animated and with great difficulty restrained from intemperance. In such subjects, Dr. V. asserts, that the free use of the lancet should precede every other remedy, and that previous vomits are rather dangerous than useful. Dr. Vetch remarks,

"With respect to Sir William Adams' treatment, in the commencement of the disease, by violent vomiting, I shall say but little, convinced as I am, that even he himself, should he ever see a case of real Egyptian Ophthalmia, in its violent and purulent stage, will not venture to place his principal trust in such a remedy."

"I proceed to examine (continues our author) the nature and the efficacy of the discovery, claimed by Sir William Adams, for the cure of opaque cornea. With respect to his present practice, I must presume, that he either adheres to his original plan of treatment by excision, which I have declared, and which I can now prove to be from his own evidence, (independent of many objections to its general application) incompetent of itself to the cure of the disease; or, that he is forced to combine with the operation those very means which it was introduced to supersede, and of which, I may venture to say, that Sir William Adams has still something to learn, both as to their value, and their proper mode of application.* I shall, therefore, in the first place, submit some general observations respecting the disease itself; and, in the second place, I shall review the statements, now published, of its practical results.

"It has been objected to the claims of Sir William Adams, that he took the knowledge of the operation from the practice of the late Mr. Saunders; I must, in justice to myself, observe, that in my Account of the Ophthalmia of the Army, printed in 1806, when it would be easy to prove that I could not have had access to the opinions or practice of Mr. Saunders, I distinctly, and prior to any modern writer, made use of the term of Granular Surface, to describe the diseased state of the linings of the palpebræ, which supervenes on purulent ophthalmia; and explicitly mentioned the bad effects resulting from the excision of the surface so diseased, and the means which I then found, and still assert, to be better adapted to the purpose of restoring the membrane to its healthy condition. My subsequent experience rendered the cure of

---

"* In the month of September 1816, Sir William Adams admitted to a patient (who, in consequence of that admission, put himself under my care) that there were but two ways of applying caustic for the cure of opaque cornea;—one was by dropping a solution of it into the eye, which he actually ordered; the other, he said, would be so violent in its operation, as to occasion excruciating pain, and endanger the safety of the eye. I refer to the case of the Honourable Captain C———, R. N. The success which immediately attended the use of caustic in this case, without causing either pain, risque, or even momentary confinement, only shows the material difference produced by the same remedy, according to the mode in which it is employed."

opaque cornea, depending on that diseased state of the palpebral linings, so much a matter of uniform result at the Ophthalmia Hospital, that long before I heard that there was such a person as Sir William Adams, I had no reason to doubt but that my success was both understood and appreciated.*

"If it be objected to these early operations that the scissors were used instead of the knife, I beg to say, that both these instruments had been repeatedly employed; and I do not scruple to assert, that, where the operation is required, the scissors are the better instrument of the two; that the surface which follows excision by them is less irritable, and less disposed to a reproduction of fungus; that there is also less risque of wounding the semilunar cartilage of the palpebræ, an accident very likely to occur in the mode of operating performed by Sir William Adams, and which I apprehend to have happened in some cases where the operation has led to a termination fatal to the organ.

"The cure of this granulated surface of the palpebræ, by means of excision, is mentioned by Hippocrates; and the disease under the names of *sycosis* and *scabies palpebrarum* is distinctly described by the succeeding authors of the Greek and Latin schools; and the cure as precisely directed by the three methods of excision, abrasion, and cauterisation. The Arabian authors are still more minute in their details respecting the treatment of opaque cornea, under the term *sebel*, nor has it been left to modern times to suggest any improvement even in the mode of operating."

"The names of the two men whom Sir William Adams omits to notice in his published report, are, William Wells of the fifty-second, and Sergeant Treble of the forty-third, regiments: these men Sir William Adams found it expedient to reject, after having kept them for a month under his treatment, on the frivolous pretext that caustic had been applied to them by the officer who succeeded me in charge of the Ophthalmic Hospital. *Of the three remaining cases, John Winter is reported to be cured, and, according to the promise given, is discharged with a pension; John Capel is dismissed with one eye 'irrecoverably lost;' and David Grey with only one eye improved, after the lapse of two years and three months.*

"I shall afford each of these cases a separate examination.

"*First.*—In relating the case of Joseph Winter, Sir William Adams states, 'that it never was my practice to examine the interior of the upper eyelids, until my return from the York Hospital in March 1812, where I had been to see his new operations;' and when (he adds in a note) '*he saw* Dr. Vetch.' On what grounds Sir William Adams has had the hardiness to advance an assertion so wholly without foundation, I am at a loss to conceive. On the

"* The change in the direction of the army medical department subsequent to the Walcheren expedition, will explain the want of support which my services would otherwise have received."

examination of, and in the application to, the inner surface of the upper eyelids, no man can have insisted more strongly than myself. I shall annex two cases; one extracted from the hospital registers, and treated by incision, in 1809; the other by an escharotic application, in 1811, as stated by the patient himself, Captain Robinson, of the eighty-eighth regiment.

"The remaining part of the assertion, which makes me appear at the York Hospital, for the purpose of seeing Sir William Adams, and his new operations, is equally erroneous; and up to the present hour I have never been in the same room with Sir William Adams, nor seen any case on which he has operated for opaque cornea.

"I am still in possession of a letter from the late director-general, expressing his dissatisfaction at my having declined an interview with Sir William Adams; together with my answer, containing my reasons for so doing, until his operations could be judged of by their *final* effects. The time to which Sir William Adams refers, is June, and not March, 1812; in which month I did accompany Mr. Weir to the York Hospital, but without seeing either Sir William Adams or his practice, farther than the former was pointed out to me at a distance too great for me to know one person from another.

"*The second case*, John Capel.—Sir William Adams says, that this man was considered by me as incurable; a statement not only contrary to truth, but inconsistent with the whole tenor of the regulations which I had established, and which, as long as I had charge of an hospital, were steadily adhered to. By these regulations, all men affected by opaque cornea, no matter to what extent, were returned, not as blind, but as recoverable for at least garrison duty, and treated accordingly. The impaired state of this man's health, and the unfitness of the situation for his recovery, sufficiently explain the length of time, during which he continued to lose, by frequent relapses, the progress gained in the intervals: when admitted, he laboured under a third attack of acute purulent ophthalmia, and was saved from the imminent hazard of losing his right eye, by the treatment immediately resorted to.

"But a more important error in the narrative of this case remains to be noticed. At the time this man was selected by Sir William Adams, it appears, by the evidence of the official report made to the Medical Board of the state of his eyes, as well as by that of a memorandum in the hand-writing of Sir William, that he was selected with the susceptibility of recovery in both eyes; and, indeed, it is not to be supposed that Sir William Adams would have made choice of a case which was otherwise. This man, however, is, in the final return, stated by Sir W. Adams to have 'irrecoverably lost' the eye, and which, he asserts, was lost under my care. The registers of the hospital afford a minute detail of the case, the evidence from which is, that the left eye was the best of the two: Sir William Adams, in his own hand-writing, states the case as one of opaque cornea, with diseased palpebral linings, and notices an inversion of the upper eyelid, but no mention is made of the left eye being different from the right. The state of this man is farther reported by my successor at the hospital, as one of simple opaque cornea, with diseased linings of the palpebræ. That Sir William Adams should lose an eye by the operation, does not surprise me; but, if the statements I have quoted are correct, his attempt to conceal the misfortune, by such a subterfuge, is what I could not expect. It is not enough for Sir William Adams to say, or rather to prove, that he did not perform the operation on the left eye, as he must be well aware that the inflammation excited by the operation in one eye, might very possibly lead to such a return of active disease in the other, as would, in the debilitated state of the organ, eventually occasion its 'irrecoverable loss.'"

We shall conclude the account with tables representing the success of the two practitioners.

| | |
|---|---|
| "Selected by Sir William Adams, on the 12th of October, 1812, five cases—(of which | 5 |
| Two were afterwards rejected | 2 |
| Two cured of one eye | 2 |
| And one cured of both—all discharged with pensions | 1 |
| Total | 5 |

"*Extract from a General Return of the Ophthalmia Depot, from the* 17*th of November,* 1807, *(the date of its establishment,) to the* 12*th of March,* 1812; *showing the result of the treatment of Opaque Cornea.*

| | |
|---|---|
| **ADMITTED.** | |
| Labouring under opaque cornea, with vision either lost or impaired | 536 |
| **DISCHARGED.** | |
| Cured of both eyes—to their regiments | 65 |
| Ditto, ditto, but transferred to veteran battalions | 247 |
| Sent to Chelsea, on account of age and other infirmities | 70 |
| Deaths, by other diseases | 7 |
| Discharged, with pensions for blindness, being two-thirds of the total loss out of 3000 cases | 20 |
| Under treatment | 127 |
| Total | 536" |

*An Essay on the Chemical History and Medical Treatment of Calculous Disorders.* By ALEXANDER MARCET, M.D. F.R.S. &c. Longman and Co. London. 1817. 8vo. pp. 181.

THE subject of Calculary Concretion is one of considerable interest, both to the physiologist and medical practitioner; and the recent improvements in chemical analysis and manipulations have given a precision and scientific character to these investigations, which, as we observed in our last Retrospect, could not be in better hands than in Dr. Marcet's.

The first section is devoted to the consideration of the "different situations in which calculi are found in the urinary passages, and the symptoms which they respectively produce." The kidneys, the bladder, the prostate gland, the urethra, are all parts in which such concretions are likely, at different times, to lodge. Long continued pain in the region of the kidneys, accompanied eventually by purulent discharge, sometimes with copious hæmorrhage, are indications that a stone exists in the kidney; but, as very justly remarked by our author, these concretions sometimes take place in this organ, and arrive at a very great size, without such marks which, *a priori*, would be imagined necessary; and it must also be recollected, that pain, hæmorrhage, and, eventually, purulent discharges, may occur from irritations independent on calculus.

"It is probably (says Dr. Marcet) during the passage of a calculus from the kidneys to the bladder, rather than during its formation, that the greatest pain is experienced. In the latter case, the pain felt in the lumbar region is rather of the obtuse kind; whilst, during the descent of the calculus into the bladder, it is sometimes most pungent, and is very apt to shoot downwards in the direction of the ureters. In either case, the disease is frequently attended with a drawing-up of the testicle, and a sense of numbness in the thigh on the affected side. The urine is generally of a deep red colour; it is voided frequently, and in small quantity at a time, and it often deposits a brick-coloured sediment. In many instances, as I have just observed, the passage of the stone through the ureter, or urethra, occasions the most acute pain, with copious hæmorrhage; yet, at other times, a calculus is discharged without the least pain, and even without the patient being aware of its passage. A thick ropy mucus is commonly voided with the urine, or sometimes, though clear when first voided, the urine soon deposits a quantity of the mucous or puriform substance, which is often tinged with blood, and remains adhering to the vessel when the urine is poured off; and the red particles, which were diffused through the urine when first voided, gradually attach themselves to the mucus, the supernatant fluid remaining nearly colourless."

The presence of stone in the bladder is, for the most part, rendered pretty certain by distinct symptoms, although these symptoms necessarily vary, both according to the size and situation of the calculus in this viscus, and also with the kind of concretion that is deposited. Dr. Heberden, we recollect, in his Commentaries, seems to consider that disordered affections of the prostate are those which, without care, are most likely to be confounded with stone in the bladder. He gives one mark of distinction, which the young practitioner will do well to attend to, viz.—that, in the prostate affection, the pain experienced on making water will be always in the commencement of micturation; while, on the contrary, it is most usually during the passage of the urine, or when the bladder is nearly emptied, that pains and obstructions are perceived in cases of calculus. Another important diagnostic mark of stone is, that the irritation which it induces does not so much affect the general health as the same degree of local disturbance from other causes. This distinction, likewise, the same venerable author points out; and we have thought it not improper here to advert to them, since what may be termed antithetical signs are more calculated to impress the minds of young practitioners than the mere enunciation of symptoms without contrast. With regard to the actual existence of stone in the prostate, Dr. Marcet well observes, that a decisive diagnostic is still wanting. Sometimes, however, as in a case related by our author from Mr. A. Cooper, the fact may be ascertained by manual examination; and these obstructing bodies, when they are in the urethra, cannot fail of being soon discovered by unequivocal marks.

The second chapter is occupied by an investigation of the comparative frequency of calculous concretions in different countries, with a view to ascertain, if possible, upon what particular circumstances of excitement the disease depends. Our author's inferences are, that no peculiarity of diet or drink, to which calculous formations and deposits have been attributed, can be justly regarded as producing any specific influence on their production. One fact Dr. Marcet's investigations served to prove; viz.—that, in hot and tropical climates, the diseases in question are much less frequent than in more northern latitudes; and hence he conceives that there may be some hitherto unobserved connexion between the excretion from the surface and the condition of the urinary organs, which favours the generation of stone. He afterwards proposes a practical deduction from this circum-

1

stance, and suggests whether, by a more than common attention to the functions of the skin, the tendency to lithic deposits might not in some measure be obviated. May we venture to add a short remark? Is it not probable that the frequency of calculi in certain districts, and the exemption in others under the same latitude, may arise from some constitutional peculiarity in the inhabitants, kept up, especially among the inferior ranks, by frequent intermarriages? These predispositions are, we know, more frequently brought into action in proportion as the mode of life is less improved, of which scrofula is the most striking instance. A note of Dr. Marcet's seems to confirm the same respecting calculous diseases in France. "Before the revolution," says he, "the average number of stone-patients was ten (instead of six). The gradual diminution of this disease since the revolution is ascribed to the improvement which has taken place in the condition of the poorer class, especially in regard to their diet."

In the third section, the different species of urinary calculi are brought under consideration; and, as this is the most important part of the enquiry, a considerable share of attention is properly given to it. Dr. Marcet first objects to that mode of classing these concretions which turns either upon their shape, size, situation, or colour; and concludes that nothing very distinctive or satisfactory can be obtained on the point of classification, without taking into account their chemical analysis, in combination with their external appearances. After a brief history of that part of animal chemistry which has reference especially to these productions; and, after noticing particularly the want of candour and justice in the French chemists, in neglecting to acknowledge their obligations to Dr. Wollaston in this particular, our author proceeds in the following words:—

"The substances hitherto discovered in urinary calculi, by the labours of the philosophers above-mentioned, are as follow—

Lithic or Uric Acid,
Phosphate of Lime,
Ammoniaco-Magnesian Phosphat,
Oxalat of Lime,
Cystic Oxyd.

To which enumeration may be added, a variable proportion of animal matter, connecting and cementing the other ingredients. It very seldom happens that these substances exist singly, and in a state of perfect purity, in urinary concretions; yet some of them generally prevail in a sufficient degree to impart to the calculi a peculiar character. And, when the mixture is such as to preclude the appearance of any characteristic form, I would (in compliance with Dr. Henry's suggestion) assume this circumstance as the distinguishing quality of an additional species of urinary concretions. Upon the whole, therefore, the different kinds of urinary calculi may be arranged under the following heads, viz.—

"1. The *lithic* calculus;

"2. The *bone-earth* calculus, principally consisting of phosphat of lime;

"3. The *ammoniaco-magnesian phosphat*, or calculus in which this triple salt obviously prevails;

"4. The *fusible* calculus, consisting of a mixture of the two former;

"5. The *mulberry* calculus, or oxalat of lime;

"6. The *cystic* calculus, consisting of the substance called, by Dr. Wollaston, cystic oxyd;

"7. The *alternating* calculus, or concretion composed of two or more different species, arranged in alternate layers;

"8. The *compound* calculus, the ingredients of which are so intimately mixed as not to be separable without chemical analysis.

"9. Calculus from the *prostate* gland."

After entering into a somewhat minute detail of the chemical properties of these several species, Dr. Marcet describes two non-descript calculi which he has met with, but which are not capable of being referred to any of the above divisions: one of these he proposes to name the *xanthic oxyd* calculus, and the other the *fibrinous*; the former being apparently composed of an oxyd, and forming a lemon-coloured compound when acted upon by nitric acid; the latter being possessed of those properties which correspond exactly to those of fibrine.

With respect to the comparative frequency of the different species of calculi, which is the next topic of Dr. Marcet's investigation, he makes the following inferences as the result of his researches.

"It would, therefore, appear, so far as depends upon the evidence of this document (a document from the Norwich collection), that the lithic calculus, which Scheele supposed to be the only species of urinary concretion, constitutes hardly one-third of the total number of stones which occur in the urinary passages; and that the fusible comes next, in regard to frequency. It appears, also, that the number of either the fusible or mulberry calculi amount only to about two-thirds of the number of the lithic calculi; and that those concretions which are evidently of a compound nature amount only to about one-half of the mulberry species. It will be also observed (adds Dr. M.) that by far the greatest proportions of deaths has been amongst patients labouring under calculi of the compound or mixed kind; and I am enabled to add, by a more particular reference to my notes, that no less than five deaths were annexed to the fifteen cases of alternating lithic acid and oxalat of lime; whilst, contrary to all expectation, the strongly characterised mulberry, with its usual rough tubercular surface, yielded a much smaller proportion of fatal cases than any other species. This result is the more curious, as it seems to show that it is not so much the mechanical irritations of the stone, as the particular diathesis of the urinary secretion, which influences the event of the operation."

The tests of discrimination in each species, upon which our author next treats, are briefly the following.—Take a fragment of the stone which you conceive to be formed of the lithic acid; expose it to the flame of a blow-pipe, and, if a lithic calculus, it will immediately blacken, emit a smoke of a strong characteristic odour, and be gradually consumed. Secondly, put another fragment of the same concretion in a glass vessel, and pour some caustic, or pure alkali, upon it, which, with the assistance of heat, will readily dissolve it. Lastly, add only a drop of nitric acid on a small particle of lithic calculus, and, adding heat, the lithic acid will immediately disappear.

The phosphate of lime calculus is to be discovered by its first blackening before the flame of the blow-pipe, but soon afterwards becoming perfectly white, and not fusing, unless by very intense heat. The muriatic acid is a ready solvent of this species of calculus. The ammoniaco-magnesian phosphat will lose its ammoniacal principle by the heat of the blow-pipe, and the remaining parts, which are a phosphat of magnesia, become opaque, and may be imperfectly fused. This calculus may be dissolved more easily in dilute acids, than even the phosphat of lime. When the fusible calculus is exposed to the flame of a blow-pipe, it melts, bubbles up, and runs into globules of a pearly appearance. This calculus, likewise, is of easy solution in the acids. The mulberry calculus is, for the most part, sufficiently obvious by its external characters. "Its most obvious chemical character is to swell out when exposed to heat, and to expand into a kind of white efflorescence, which, when brought into contact with paper stained with the juice of violets, and slightly moistened, turns it green." The waxy appearance of the cystic oxyd stone, its peculiar smell when heated, and its easy solubility, both in acids and in alkalies, constitute its principal features of distinction.

Before engaging in the consideration of the treatment of calculous disorders, Dr. Marcet occupies a section of his work with noticing "some other kinds of animal concretions, not belonging to the urinary passages, both in man and other animals." Of these, he particularizes concretions in the salivary glands, which, according to Fourcroy and Bostock, "consist of phosphat of lime, with small portions of animal matter;" intestinal concretions, which, in the human subject, are rare, and possess very little distinctive character; "calculi from the intestines of quadrupeds, which may be generally stated to consist of the ammoniaco-magnesian phosphat;" urinary concretions of animals, which generally differ from those of the human subject in containing no lithic acid, and in consisting principally of carbonat and phosphat of lime, cemented by animal matter. Indeed, we are told that the lithic acid has never been discovered in any animal concretions, except in man, "till Dr. Prout analysed the excrement of the boa-constrictor, and found that substance to yield upwards of nine-tenths of its weight of lithic acid, and to contain ammonia." With respect to gouty concretions, these have long been ascertained to consist of lithic acid and soda. Biliary calculi, which are the last substances mentioned by our author, are constituted mainly of the substance called, by Fourcroy, *adipocere*, from being a kind of *sui-generis* principle, seemingly between wax and fat.

In the last chapter, Dr. Marcet discusses the treatment of calculous complaints. This discussion he introduces by the following candid admissions and philosophical remarks.

"On my entering upon this subject, I think it necessary to premise that, in endeavouring to apply chemical principles to this branch of medical practice, no reasonable expectation can be entertained that calculi, lodged in the urinary organs, and already too large to be discharged by the natural passages, can be actually dissolved by any mode of internal treatment. The only benefit which we may, with any confidence, expect from medicine in this disease is, either to prevent the increase of calculi already formed, or, what is still more important, to guard the constitution of those who are subject to the disorder, against the prevalence of the particular diathesis from which it arises. But, although we cannot materially affect large concretions by medicines, on account of the powerful resistance which the cohesive force of such calculi, and the small extent of surface which they present in proportion to their mass, necessarily oppose; yet there certainly are cases in which some impression may be made upon small calculi, or gravel, so as to blunt their sharp edges, and enable them to be discharged by the urethra with less difficulty or inconvenience. At all events, since, in attempting to remove calculi, we have to contend against unorganised bodies, which, though contained in living parts, do not obey the laws of the living principle, it may be fairly concluded, that, unless surgical aid be resorted to, it is, in a great measure, from chemical principles that our views of treatment must be derived."

It is well known that doubts have been entertained and expressed as to the possibility of any direct influence upon the urine, or urinary concretions, by substances taken into the stomach; and an inference has been adduced from such doubts, of the fallacy of those expectations which calculate upon the corrective virtue of reputed solvents, or remedies for stone and gravel. But it is an actual fact, resting upon the most authoritative testimony, that "alkaline medicines will not only deprive the urine of its acid properties, but will render it decidedly alkaline." This fact was proved, in a very satisfactory manner, by the Bishop of Landaff, who, while taking lime-water for a cure of stone in the bladder, poured his urine, every morning and evening, upon a piece of human calculus, weighing thirty-four grains, by which, in the space of four months, it was reduced to three pieces, weighing in all six grains. But it may be said, that this solution might have been effected by common urine. To prove this not to be the case, the Bishop caused to be daily poured, for two months, upon a piece of the same calculus, the urine of a person who drank no lime-water; at the end of which time, the substance was found to have increased instead of undergoing diminution:—a clear demonstration this of the principle contended for, viz.—that certain substances, taken into the stomach, actually produce a direct chemical change upon the urinary secretion. A case, published more recently by Dr. Bostock, serves indubitably to establish the same assumption; and "Mr. Brande has shewn that alkalescence in the urine is produced within a few minutes after taking the alkalies, whether they be in a caustic or pure state."

"With regard to the acids, (says our author,) the question is not so easily resolved. For, as the urine is naturally acid, and especially contains portions both of the muriatic and sulphuric acids, which are those commonly used as medicines, any small increase of either of these acids in the urine, in consequence of their being taken into the stomach, cannot be so readily ascertained. It is, however, stated by some chemists, and in particular by Mr. Brande, that acids taken into the stomach are actually capable of being conveyed into the bladder; and this he has more especially endeavoured to ascertain, by experiment, with regard to the carbonic acid.

"Unfortunately, however, (our author adds, in the same spirit of candour which is exhibited in the extract above made,) although alkalies do certainly, and acids may possibly, reach the urinary passages, yet experience has shewn that the quantity of either, thus conveyed through the circulation, is so small, that very little, if any, impression can be made on large pre-existing calculi, with whatever freedom or perseverance these medicines may be used. But there is abundant evidence to prove that we are able, in many instances, to produce an effect sufficient to check the prevailing diathesis, and also sometimes to bring on a calculous deposit, depending upon an opposite state of the system; a change which I have myself repeatedly witnessed."

Even were it proved that neither acid nor alkali could be conveyed directly to the urinary passages, and thus influence calculous disorders, the lithontriptic virtue of either might still be maintained, upon the ground of their correcting that disposition in the stomach and first passages, upon which calculary deposit so materially depends; and it must be always expedient to ascertain, when we are capable of doing so, the chemical composition and nature of the particular concretion, against which we are instituting our remedial processes. When the acid solvents, or correctives, are required, Dr. Marcet tells us that from five to twenty-five drops of the strong muriatic acid, two or three times a day, sufficiently diluted with water, are the doses which he usually administers; and, as to the alkalies, soda-water is a very convenient form of administering this species of medicine; and, when this cannot be procured, "from five to twenty grains of carbonat of soda, whether in a state of subcarbonat, or in that of neutral crystallised carbonat, may be taken two or three times a day, dissolved in a little water, —a remedy which generally produces relief, without occasioning any obvious inconvenience."

In this division of his treatise, Dr. Marcet replies to the objections which have been urged against the direct lithontriptic powers of alkalies, on the ground that the mild alkalies, or those impregnated with carbonic acid, have been found equally, if not more, efficacious than the pure or caustic substances of this nature; whereas, it is these last which are the solvents of lithic concretions out of the body. The objection does not take into the account, that the carbonic acid of the mild alkalies, meeting with acid in the first passages, becomes neutralised and expelled; and thus a double operation is effected, both solvent and preventive, since the correction of the lithic condition of the stomach is, as we have just hinted, an important principle in the treatment of calculous disorders: and, indeed, it is further maintained by some, that "carbonic acid is of itself capable of penetrating into the circulating fluids, and actually reaching the bladder, in its uncombined state, so as to act as a solvent upon the calculi contained in it." This last supposition, however, Dr. Marcet considers as having by no means been fully verified.

Magnesia has been recently proposed, and very extensively used, as a substitute for alkaline lithontriptics, and our author allows it to be a useful addition to the medical treatment of calculous disorders; but it may prove actually deleterious when prescribed without a knowledge of the nature of the existing calculus, as in the ammoniaco-magnesian phosphat; and it is well known that serious inconvenience has, in some cases, arisen from the accumulation of large masses of this substance in the intestines, when it has been too copiously employed. These considerations ought to induce caution and discrimination in the application of this remedy.

After making some remarks on the alkalies, as medicines capable of allaying irritation, as well as correcting lithic dispositions, our author goes on to notice the difficulty of treatment, from the alternation and complication of calculous deposits; the means proposed by Fourcroy, but which have not been adopted in this country, of injecting substances into the bladder, for the purpose of ascertaining the nature of the concretion in the bladder; and the suggestion of Dr. Wollaston, that, in the oxalat of lime concretion, its increase might be prevented by avoiding saccharine materials of diet. He then proceeds to state, that purgatives may probably be more efficacious than is generally imagined in correcting calculous tendencies; and that the regulation and increase of the cutaneous discharge may also be regarded as an important principle in the treatment of calculous disorders, upon the principles before adverted to. With respect to diet, Dr. Marcet seems of opinion that every thing which can be considered as an established principle in this respect is referable to excess, or to the avoidance of those articles of food or drink which are known to increase or produce acidity in the stomach. On this head, we think our author seems to lean rather too much towards those modern simplifications taught by the "digestive-organ theorists." Turpentine, combined with opium, is a medicine, we are told, of considerable auxiliary use in calculi; and the treatise is concluded by a few remarks on alkaline and acid injections into the bladder,—a practice which Dr. Marcet thinks might be prescribed advantageously, to a greater extent, and more generally than has hitherto been done.

We regard this treatise as very creditable to the author's abilities and acquirements. The plates are most exquisitely executed; and, although they add, of course, considerably to the expense of the publication, they certainly increase its value in the same proportion.

*Medico-Chirurgical Transactions, published by the Medical and Chirurgical Society of London.* Vol. VIII. Part II.—Longman and Co. 1817.

*Observations on the Nature of some of the Proximate Principles of the Urine; with a few Remarks upon the Means of preventing those Diseases connected with a Morbid State of that Fluid.* By William Prout, M.D.

The number of men, on whose experimental accuracy we can depend, throughout Europe, is small compared with the number of experimenters. It is, therefore, fortunate, when more than one of the same description are about the same time engaged in similar pursuits, and peculiarly satisfactory, if the result from the enquiries of each should be similar.

In this paper, Dr. Prout confines his attention to urea, saccharine matter, and the lithic or uric acid. "The other principles, particularly the phosphates and oxalic acid, are omitted from the uncertainty which still hangs over their nature."

Hereafter, we mean to devote a considerable part of some future Number to the late improvements in chemistry. We shall then enter minutely into the combinations detected by the author, and the manner in which he conducted his experiments, which will be further illustrated by a diagram of his instrument. At present, he considers the enquiry as imperfect in his own hands; we shall only, therefore, offer the general conclusions as subsidiary to Dr. Marcet's work, adding a very few remarks of our own.

"*General Conclusions.*—1. The atomic theory, or theory of definite proportions, holds good in all these instances. A circumstance which renders it probable, that this will afterwards be found to be the case in all substances capable of crystallizing or forming crystalline compounds, both in the vegetable and animal kingdoms.

"2. The above compounds appear to be formed by the union of more simple compounds, as urea of carburetted hydrogen and nitrous oxide, lithic acid of cyanogen and water, &c., circumstances which render it almost certain that their artificial formation falls within the limits of common chemistry.

"3. The remarkable relation found to subsist between urea and sugar, seems to explain, in a very satisfactory manner, the phenomena of diabetes, which may, in fact, be considered to consist in a depraved secretion of urea. Thus, the weight of an atom of sugar is just half that of urea: the absolute quantity of hydrogen in a given weight of both is equal, while the absolute quantities of carbon and oxygen in a given weight of sugar are precisely twice those in urea.

"4. Lithic acid is a substance quite distinct from urea in its composition. A fact, which explains an observation I have often made, that an excess of urea generally accompanies the phosphoric diathesis, and not the lithic. I have several times seen urea so abundant in the urine of a person where the phosphoric diathesis prevailed, as to crystallize spontaneously without being concentrated by evaporation, on the addition of nitric acid.

"I shall forbear at present to notice other interesting circumstances suggested by the present enquiry, lest, in this early stage of it, they might be considered as visionary and hypothetical. These analyses, however, appear to me to afford glimpses of laws that will hereafter be found to influence the whole system of Nature's operations.

"SECTION 2.—*Some Remarks upon the Efficacy of General Remedies, and especially Purgatives, in ensuring a Healthy State of the Urinary Secretion, and thus in preventing Calculous Affections.*

"The observations I have to offer on this head are not to be considered as deductions from those contained in the preceding section. Practical facts in general have not been deduced from physiological knowledge, but have usually been the result of accident or blind experiment. Even in the present improved state of anatomy and physiology, we cannot pronounce *a priori* upon a single effect which any new substance will produce upon the animal economy. Such reflections are, doubtless, mortifying to the cultivators of these branches of knowledge, especially when aggravated by the sneer and *cui bono* of ignorant empiricism; but the series of cause and effect, which at present separates physiological from practical knowledge, cannot be *infinite*, and by gradual approximations from either extreme, the whole must sooner or later be explored, and 'reason will become triumphant.'

"*Uromancy*, or the practice of judging of the nature of disease, from the appearances of the urine, seems to be almost as old as medicine itself. The opinions of the ancients, however, on this subject, were vague and often ridiculous, and it is to modern chemistry, entirely, that we owe the means of making accurate observations upon the urine. Some years have now elapsed since I more particularly turned my attention to the pathology of this secretion, and one of the earliest observations I made (which has, doubtless, been often made by others) was the striking effect produced by a common laxative, in restoring my own urine from an unnatural and turbid state to its proper colour and transparency. After having made this remark, it required but little reflexion to arrive at the conclusion, that the cause, whatever it might be, which rendered laxatives necessary, contributed chiefly to produce this unhealthy state of the urine; and, from the attention which had been lately paid in this country to the diseases of the digestive organs, it was easy to refer this common cause to derangements in the functions of these organs. But, being aware of the close affinity which subsists between urinary deposits and urinary concretions, the question here naturally occurred, if purgatives have the power of removing urinary sediments in common cases, would they not have the power of removing them in extreme cases, or in gravelly and calculous affections? From want of proper opportunity of verifying these speculations, they were almost forgotten, till I was introduced to Dr. Scudamore, who I found entertained similar views, and had prosecuted the subject much further than I had done. This gentleman, in his late publication, has, indeed, anticipated most of what I had to say upon the subject; a circumstance that will render it unnecessary for me to enter into details here, and limit my views chiefly to the extensive circulation of general principles only, through the medium of this society.

"Vitiated secretions of every description must be the result of general or of local causes, or of both united. But, when we reflect how little liable the secreting organs are to be affected, and how seldom, in point of fact, they are affected, except through the medium of the general health, we are naturally led to look here for the primary cause of their derangement. The inference is obvious. Remedies, no matter of what description, that have a tendency to restore the general health, must have a tendency to insure the due performance of all the bodily functions, and secretion among the rest. I need not enlarge here upon principles which are well understood, and the elucidation and application of which are justly ranked among the greatest discoveries of modern medicine; but shall merely observe, that, by paying proper attention to the general health, and especially to the functions of the stomach and bowels, I have, in numerous instances, witnessed the speedy removal of urinary deposits, and the complete restoration of this secretion to its natural appearance and properties. This has been remarkably the case in children, in whom the phosphoric diathesis most generally prevails. The remedy employed has been, for the most part, a combination of calomel and rhubarb, in connection with which, others were occasionally exhibited as circumstances rendered it necessary. In adults, as is well known, both the phosphoric and lithic diatheses prevail, and often alternate in the same person. I have, however, generally, seen both equally yield to the same principles of treatment, and, sometimes, even to the same remedy, and am disposed to think, therefore, that they are more intimately connected than commonly imagined. Some differences, however, must be admitted to exist between causes which can produce such different effects, though I must confess myself unable, after a good deal of attention to the subject, to point out, or even to offer an opinion of their nature. As to particular remedies, they will readily occur to the practitioner who keeps the above-mentioned principles in view. I may, however, observe, that, when laxatives have been particularly indicated, I

have been accustomed to exhibit a combination of the pil. hydrarg. with aloes, or the ext. colocynth. c. with the best effect. Remedies determining to the skin and kidneys are often useful adjuncts, and a regimen in strict unison with the same general principles should be adopted.

"I need hardly remark, that the above observations are almost entirely confined to diseases of the urine, while as yet they are merely constitutional, and have not produced local disease, or actual calculus. To the treatment of such unfortunate cases, I have nothing to add to the little already known. When a calculus is once formed, its further enlargement is, probably, a common chemical process, and will proceed, whether the urine be healthy or not, for all urine naturally contains the ingredients most commonly met with in calculi. Something, however, may be possibly done by general remedies in retarding its growth; but this will only prolong the patient's misery, who had much better, therefore, submit at once to the operation, and, by subsequent attention to the above principles, prevent the future formation of another.

"I have had no opportunity of applying these principles to diabetes: but, when this disease is fairly formed, it seems to become, in a great degree, of a local nature; hence, probably, they will be inapplicable.

"I shall close these remarks with a few observations upon the *chemical* mode of treating calculous affections.

"The principles of the chemical treatment of calculous affections are well understood, and have been very lately ably explained by Dr. Marcet. Acid and alkaline remedies are, doubtless, often productive of much good in diseases of this description. Alkaline remedies, in particular, are universally admitted to have the property of lessening that excessive irritation commonly attending such affections. From all, however, that I have been able to observe, I am obliged to confess, that it appears to me, that the good effects, both of acid and alkaline remedies, cannot be altogether satisfactorily explained upon chemical principles. Indeed, Dr. M. admits this to be the case in a certain degree, and I am also borne out in this opinion by many facts on record: thus, Berzelius informs us, that he exhibited large doses of the sulphuric, phosphoric, and acetic acids, in succession, to a patient whose urine was alkaline, and deposited the phosphoric, but without the least good effect, till the phosphoric acid was given in such quantity as to prove laxative, when 'the urine became acid, and deposited uric acid, which continued as long as the laxative effect continued, and no longer, although the dose remained unaltered.' Alkaline remedies also are stated by Dr. Marcet, to 'often allay the irritation of the bladder, and promote the flow of urine, even when, from the chemical composition of the concretions, they can be of no use as solvents.' So also magnesia, of which so much has been said, seems to me to produce little satisfactory good, unless its laxative operation be secured. But, when we reflect, that all urine (except, perhaps, in extreme cases of diabetes) contains both lithic and phosphoric acid, although only one of the diatheses generally prevails at the same time, the conclusion is probable, reasoning merely *chemically* upon the subject, that the exhibition either of acid or alkaline remedies may produce harm as well as good: and, if we take into account, also, the capricious nature of secretion, and the frequent alternation of these two diatheses in the same person from unknown causes, it becomes an exceedingly difficult task to adjust the remedy to the disease; and the *chemical* probability will be, that the disease will ultimately be increased, instead of diminished. Lastly, the *object* of the chemical practitioner is, at best, but of a secondary description, namely, to prevent the effects of disease rather than to remove it. From these and other circumstances, therefore, which might be mentioned, I have been induced to consider chemical remedies as palliatives only, and to explain their acknowledged good effects even in this way, rather upon their general than their chemical operation; but this opinion, as well as the others advanced above, I submit to the medical world with the greatest deference."

It is impossible not to admire the modesty, the industry, and, we doubt not, the accuracy, of this meritorious philosopher. We confess ourselves particularly pleased with the manner in which Dr. Prout informs us he has been accustomed to exhibit the Pilul. Hydrarg. in combination with aloes or Extr. Colocynth. c.; and also his candid remarks that some other remedies may owe more to their laxative than their chemical properties. In an art so purely experimental as medicine, it is of the utmost consequence to treasure up facts, when delivered to us from men of sound judgment and accurate observation. On this occasion, we wish particularly to call the attention of our brethren to a due consideration, whether gentle purgatives (as Dr. Marcet, with Dr. Prout, seems to hint) may not be attended with all the advantages ascribed to what are called alterative mercurials. Our own experience strongly favours this conclusion; and we shall show that it stands on an authority more venerable than is suspected by those who undervalue medical reading.

"Happening afterwards to recollect the great commendations which some persons have bestowed on the seed of the ash-tree, for its stone-dissolving, or stone-breaking, virtue, I imagined that, if the seed had so much virtue, the manna thereof might probably have more. For the manna which comes to us, according to Mr. Ray, and other earlier writers, is neither an aërial honey, nor a kind of heavenly dew, but rather a liquor oozing from the leaves, branches, or trunk, of the Calabrian ash-tree; of the truth of which Mr. Ray was further satisfied, whilst he was on his travels in Italy, by a physician, who frequently gathered manna from the branches and leaves of these trees, first closely covered with linen cloths. Accordingly, to make the trial,* I dissolved two ounces and a half of manna in a quart of whey, and drank it, and took a little lemon-juice between whiles, as well to make it operate more speedily, it being ordinarily a slow purgative, as to render it more agreeable to the stomach. It is hard to express the ease I perceived in the region of the kidnies from this medicine; for, though the pain was not continual before, yet I felt a troublesome weight. Encouraged by this good success, I took this purgative every week on a set day, for some months, and found a manifest amendment after every purge, till at length I could bear the shaking of a coach when the horses went apace; and, indeed, continued free from this symptom till last spring, at the beginning of which it returned, occasioned by my having had the gout severely all the preceding winter, and my inability to motion, which made me indulge rest, and use less exercise than usual. And now I doubted whether I should have recourse to purging again, as finding that the mildest purge certainly occasioned a fit of the gout, because the whole substance of my body, in these latter years, had, in a manner, degenerated into nourishment for this distemper. But, at length, it came into my mind, that I might safely resume my former method of taking manna once a week, provided I took an opiate in the evening after the operation, to quiet the tumult raised by the purgative. Accordingly, in the morning I drank two ounces and a half of manna, dissolved in a quart of whey; and, at night, took sixteen drops of liquid laudanum in small beer; and repeated the manna and laudanum, in this manner, twice a week, for three weeks running. But afterwards I took the manna only once a week, because it had discharged such plenty of foul humours as to leave little fear of the gout. And my reason telling me, that, if manna was possessed of any stone-dissolving or stone-breaking virtue, its efficacy, on which I depended, must needs be lessened, in some measure, by so powerful an astringent as laudanum is, I thought it best to omit taking the opiate, as I only purged once a week."—*Sydenham*, p. 532-533.

By such a remedy it is not unreasonable to conclude, that, action and secretion being excited in different organs, a disease may cease in those which were previously affected, and that the parts may be protected from the irritation of the previously formed stone, in the manner described by Dr. Marcet. This is only one of the considerations we wish to impress on our readers. Whatever alters action in a chronic disease is likely to supersede such diseased action, and consequently to restore healthy action. In this manner only, alterative doses of mercury produce their salutary effect, and would be unobjectionable, if it were not for the high irritability induced by a frequent repetition of such a remedy. Laxatives are unattended with such danger, and, in our opinion, are more certain in their good effects.

* See his Catalogue of English Plants.

*Commentatio de Pathologia Lienis, Observationibus per Anatomen institutis, indagata, ad illustrandam Physiologiam ænigmatici hujus visceris.* Auctore C. L. SCHMIDT. Gottingae. 1816.

THE object of this dissertation is to illustrate the physiology of the spleen, by observations relating to its structure and diseases. An exact description of the situation, form, usual magnitude, and weight, of this organ is first given; also of its lymphatic vessels, nerves, and especially of its great artery and vein.

The diseases of the spleen have been neglected or mistaken, from being confounded with those of other parts. The vomitting of blood, dyspepsia, difficulty of breathing, have been considered as symptoms of different diseases; and the unfrequency of a fatal termination has prevented the truth from being discovered after death. Yet the derangements of the spleen are so common, that it is more rare to find it in a sound than in a diseased state. The inflammations of this organ are divided into *acute* and *chronic;* the acute inflammation varying as to its extent, through the whole, or confined to a portion, is either *universal* or *partial:* and, as other viscera are occasionally inflamed at the same time, it is also to be considered as *simple* or *complicated.*

As the inflammation of an abdominal organ is rarely confined to that organ during its whole course, it will be difficult to distinguish the acute inflammation of the spleen from that of other parts, except in the earliest stage: nor is it of much consequence. It appears that persons of both sexes and of all ages are liable to the complaint, but it most frequently occurs in females, who are affected with a deficiency or entire suppression of the menstrual evacuation; that the melancholic temperament is more disposed to it than the sanguine; and that it is observed most frequently in humid and warm countries, such as Hungary, Bengal, and Italy.

The occasional causes are such as the application of cold to the feet or breast, a concussion, a blow, a fall, suppression of the menstrual or hæmorrhoidal fluxes, or of any other customary discharge of blood.

Inflammation of the spleen is preceded by the following symptoms:—The patient becomes dull, morose, and inclined to anger; there is a sense of uneasiness in the præcordium, an aversion to food, and a difficulty in digesting it; the countenance becomes pale, livid, and often, from the sympathetic affection with the liver, of a yellow colour. To these symptoms are added chills, followed by heat; the patient is uneasy, and cannot rest either on the right or on the left side, but is most easy in a supine posture, and yet unable to sleep. In consequence of the little irritability of the spleen, these symptoms are protracted to the fourth, and even to the seventh day, which is not observed to happen with the other abdominal viscera.

Symptoms of the disease:—1. The most constant symptom, and one which leads with most certainty to distinguish the disorder of this organ, is an *anxiety and straitness in the præcordium*, with difficult respiration, often conjoined with a cough without expectoration. This may be supposed to arise from a sympathy of the lungs, rather than inflammation of the diaphragm, or pressure from the enlarged spleen. 2. The patient complains also of internal *heat, tension and pains* in the left side, which sometimes extend through the whole region of the abdomen, or shoot through the diaphragm, and into the left shoulder: these pains are increased by pressure, and are pulsatory, pungent, and burning in various degrees. 3. *Pulsation* and *palpitation* in the left hypochondrium and on the back. These symptoms are sometimes violent. Tulpius relates a case "where he could distinctly hear the stroke of the beating spleen, at the distance of thirty feet." 4. The pulse in the left side is sometimes suppressed, often intermittent, week, and not quick. 5. *Lassitude* and loss of strength. 6. *Watchfulness*, with delirium. 7. *Dyspepsia*, *anorexia*, *vomitting* of green billious matter, and sometimes difficulty of urine from affection of the kidney or bladder. 8. *Swelling* in the region affected, representing the form of the spleen. 9. *Fainting*. 10. *Bleeding* from the nostrils, at the height of the disease, which, in due quantity, terminates the complaint; and the more profuse it is, the more does it alleviate the fever, pain, and vomiting of blood. Sometimes, however, it is so excessive as to prove fatal. It is wonderful, that from Hippocrates to the present day, this should be observed to occur from the left nostril. 11. The most remarkable symptom attending this disease is the *bloody vomiting*, which most authors have considered as peculiar, and have designated by various names. Hippocrates called it melæna. Schenkius, dejectiones nigrae. Guaronius, morbus niger. The ancients, bilis atra; for they supposed it to consist of true bile. Marcus was the first physician who gave sufficient weight to this symptom; he even says that every bloody vomiting depends on the spleen; and that an inflammation of this organ does not occur without it. But, surely, if this phenomenon is so constant, we ought to meet with it more frequently, since the spleen is so often disordered.

The usual course of the complaint is this: The patient, after being unwell some days, and vomiting mucus, the gastric fluid, and bile, is attacked with fainting and vomiting of bloody, frothy, and sometimes black-coloured liquids; and, soon after, the intestines, which were previously confined, become relaxed, and emit substances coloured by black blood, which is sometimes thought to proceed from a hæmorrhoidal complaint. The vomiting of blood is, under common circumstances, to be considered a favourable and critical evacuation; yet sometimes it proceeds to a fatal excess.

Dissections of dead bodies shew that the spleen is inflamed, and even gangrenous; and the surrounding organs partake of the inflammation.

The author afterwards treats of the slow inflammation of the spleen, of its displacement, change of texture, deficiency, extirpation, and rupture.

The conclusions, drawn from a multitude of anatomical and pathological facts, are these: that the spleen is an organ peculiar to red-blooded animals; that it is of the greatest importance in preparing and mixing the blood; and that its action is of as great importance to the liver as that of the lungs to the heart.

In support of which opinions, the following arguments are summarily adduced:

"1. The peculiar influence of the spleen on the blood is shewn,

"*a.* By the consent (sympathy) observed with the liver, heart, and lungs, in diseases of the spleen.

"*b.* By the increased quantity of blood in the whole body, when the bulk of the spleen is increased.

"*c.* The many secondary affections of the system, conjoined with diseases of the spleen.

"*f.* The experiments of Home exhibit the singular influence of the spleen on the blood; since heterogeneous particles, as, for example, of rhubarb, were separated by the assistance of the spleen. Hence a dispute, whether the spleen contributes to the formation of bile; which seems probable, since it appeared, in the dissection of animals who had suffered extirpation of the spleen, that bile was colourless.

"2. The business of the spleen, as a venous lung, is demonstrated by the following facts,

"*a.* The operation of diseases on the spleen, as on a vein; for example, its enlargement, ossification, cartilaginescence.

"*b.* The change of colour in the spleen with advancing years.

"*c.* The increase of the venous system, observed on the increase of the size of the spleen.

"*d.* The great consent of the spleen with the lungs.

"*e.* The greater size of the spleen in animals inhabiting marshy regions.

"*f.* The structure of the spleen being a venous character.

"*g.* Glands in the spleen, which may be compared to the bronchial glands.

"*i.* Enlargement of the spleen, often observed before the menstrual evacuation."

Whatever may be thought of the author's deductions, we must give him credit for contributing to the improvement of medical science, by assembling and arranging a multitude of facts relating to the structure and diseases of this *enigmatical viscus.*

The work contains much learning and diligence. We are by no means satisfied with the author's theories, but conceive they may lead us to trace the progress, if not the cause of some diseases which have hitherto baffled all our inquiries. We trust he would not make the bold assertions contained in the paper without ample proof and satisfactory experiments. From an acquaintance with the true characters of English pathologists, we naturally look first for their accounts; but, on this enigmatical viscus, as it is here called, we meet with no information in Cullen, and scarcely any thing beyond prudent caution in Bailie.

---

*Remarks on the Medical Care of Parochial Poor, with a few Observations on the Improvement of Poor-Houses, and on the necessity of establishing small Infirmaries in Populous Towns.* By JOHN C. YEATMAN, Esq. Member of the Royal College of Surgeons, and Surgeon-Extraordinary to his Royal Highness the Duke of Gloucester. 8vo. pp. 34. London: Longman and Co.

THE importance of this subject, and the manner it has so lately attracted legislative attention, induces us to take early notice of whatever relates to the management of the poor We have several times remarked, how much the care of the sick and the remuneration of the medical attendant are implicated in the question. If the diseases of the poor are early attended to, the probability is, that they may be soon restored to a capacity of providing for their family. Even the children, by an early attendance, may be visited with a short, instead of an expensive, chronic complaint. Add to this, there is no class of the community better acquainted with the real condition of the poor than those who see them in their domestic habits, and such is the case with medical men.

But the wealthy are by no means aware how much they are interested in the proper remuneration of the parochial medical attendant. By a just return for their services, men of talent have encouragement to remain in remote parts, where the scanty population obliges families of the first rank often to suffer irreparable mischief before they can procure the assistance of one on whom they have sufficient confidence. A close attendance on the parochial poor forms, also, a very valuable school to an apprentice or an assistant, affording them the only insight they can expect into an art for the most part practical.

We shall select some of the most useful passages from this tract, and conclude with those objections which, we hope, the author will receive as the result of long and repeated consideration on the subject. The following is part of an extract from Dr. Burrows's reflections from the number of communications he received, when president of the medical association.

" 'In relating various abuses which affected their interests, there was one in which all the country practitioners were nearly uniform; and that was the gross medical neglect of the parochial poor.

" 'In most parishes, the medical attendance is *farmed*, as it is termed: that is, a contract is made by the parish officers for attendance on paupers, at a certain sum per year. This contract is entered upon at Easter; previously to which, notice is given to all the *doctors* in the vicinity to send in their proposals: accordingly, all the regulars, who think it worth their notice, and irregulars, make their tenders; and he who offers the lowest terms, is appointed the parish *doctor* for the ensuing year.

" 'In parishes where any person of education and character resides, who condescends to enter into parish affairs, this abominable practice does not often obtain.

" 'Five pounds per annum, for medical attendance and medicines, is a liberal salary, where the casual poor have averaged from sixty to a hundred; and, even in parishes inhabited by persons of property, and who would blush to be called inhuman, I have known forty shillings only allowed; and, of contracts for medicine and attendance, at two shillings a-head per annum. This is no exaggeration. I have abundant and irrefragible evidences in my possession to support these allegations.' "

" The *casual* poor are those who belong to a different parish from that in which they reside; they have, therefore, no legal claim to local medical relief: and, to procure an order for it from their own parish, requires a *negociation of no small difficulty* between the pauper and overseer; the latter of whom generally cut it short, by requiring him to be brought home for assistance. But, as this would either endanger his life,* or deprive himself and family of that employ which kept him and them from the poor-house, he is constrained to give up all thoughts of parochial aid, and to rely on the mercy of heaven in sending him the good Samaritan, and 'the Ladies Bountiful.'

"Last year a friend of mine attended more than two hundred paupers belonging to various parishes, and, for those cases requiring prompt and instant attention, he requested a trifling remuneration. He took the trouble of stating the circumstances which prevented him, in each case, from applying for an order for attendance; but no answer was returned."

"Before me is a copy of 'Instructions to Regimental Surgeons, for regulating the Concerns of the Sick and of the Hospital,' &c. signed by the Inspector-general of Army Hospitals, and enjoined by his Royal Highness the Commander-in-Chief, bearing date January 1, 1808. And, at page 40, the regulated allowance to country practitioners is to the following effect:—'When a detachment is without a regimental assistant, and is not within reach of any military surgeon, the country practitioner may be employed. —The regulated allowance has been at 1d. per man per week, for medicines and attendance; but, when the number is under fifty, and the contract cannot be made for that sum, it is allowable to give 6d. per month.' And again, 'When, from the pressure of the moment, on a march, on sick furlough, or with recruiting parties, such agreements cannot be made, the country practitioner will be allowed to charge his medicines at a price suited to such class of patients.'

"The terms above proposed, are at the rate of 4s. 4d. and 6s. per man, per annum, and, for small bodies of men, country practitioners are remunerated in a manner corresponding with the description of persons for whom their assistance may be required.

"Country clubs, consisting of from fifty to several hundred members of the manufacturing and labouring classes, pay their surgeons from 3s. to 5s. per man per annum.†"

---

"* A sick pauper was driven in a cart into the town of Castle-Cary, by the overseer of a distant parish, and, upon his arrival, breathed his last.

"'A pauper, (says the Evening Mail of the 20th instant,) was lately brought into Salisbury, from Farmborough in Berkshire, more than fifty miles distant, in an open cart, whilst labouring under a disease of which he died two days after his arrival there: —A verdict of wilful murder is found against the overseer, who is committed for trial.'"

"† It must be confessed, that there are many instances in which false economy actuates those bodies; while practitioners demean themselves, and injure the profession, by taking a less price than 3s. per man per annum."

Taking the above as his general means of calculation, the author proceeds to detail the sum to be fixed, according to the population of each district. In this, we cannot well follow him, but those who are most interested in the question, will be in possession of the pamphlet. For midwifery, he proposes, that females should be instructed and properly certificated. In his subsequent suggestions for the improvement of poor-houses, much novelty cannot be expected.

A chapter follows, on the necessity of establishing small infirmaries. In this, we meet with one very judicious proposal,—that, till the districts are enabled to collect sufficient funds for building infirmaries, they should avail themselves of the empty houses which abound in most remote parts of the country. For our parts, we could wish that such resorts should not be entirely temporary. Large hospitals have a magnificent appearance, but they are with difficulty kept in order; and, if the wards are large, there is always danger of their being crowded; the consequence of which we need not dwell upon. If a number of smaller houses were scattered about different parts of the country, each under the inspection of some prudent woman, deserving such an occupation, (and these abound in every parish,) there would be an easy receptacle for the casual poor, and the nearest Lady Bountiful might attend to the conduct of the whole. This would soon become a fashionable employment, insomuch, that instead of any danger lest it should be neglected, we should only be fearful of too great an indulgence of that fondness for patronage, which is, at present, so generally prevalent.

The sum expended in building a county infirmary would often supply the inhabitants for the first ten years with better accommodations, under a more manageable economy, than any of the larger establishments can boast; and the attention to the concerns of each receptacle, would devolve on one or two individuals, or their families, by which means, what would otherwise be the business of all, will not be neglected by all, or left to the management of those whose whole time would be insufficient for overlooking so large an undertaking as a county hospital. In the meanwhile, the intercourse between the various classes of society would be more easy: an event which would be the best means of cultivating a mutual good opinion, by the allowance each would make for the failings, in a certain degree, attached to their different conditions, and the consequent temptations to which each are exposed.

